# Incidental findings in the oncology patient: pancreas and spleen

**DOI:** 10.1186/1470-7330-14-S1-O43

**Published:** 2014-10-09

**Authors:** Richard M Gore, Robert I Silvers

**Affiliations:** 1 Department of Radiology, North Shore University Health System, University of Chicago, Pritzker School of Medicine, Evanston, IL, USA

## 

Recent advances in multidetector CT, magnetic resonance imaging, and ultrasound have led to the detection of incidental pancreatic and splenic lesions that in the past remained undiscovered. These incidentalomas are unexpected, asymptomatic abnormalities discovered serendipitously while searching for other pathology. These incidental lesions have created a management dilemma for both clinicians and radiologists, particularly in the oncology patient in whom any mass, clinical or subclinical, warrants further evaluation.

Strategies for optimizing patient management have emerged in terms of deciding which of these incidentalomas can be ignored, which can simply be monitored over time, and which lesions require more aggressive workup. Subjecting the patient to unnecessary testing and treatment carries its own set of risks that can result in an injurious and expensive cascade of imaging and intervention. The exhaustive evaluation performed in some patients reflects the unwillingness of many physicians to accept uncertainty even in the case of a very rare diagnosis. This unwillingness is in part driven by varying algorithms with regard to diagnostic and treatment strategies, fear of potential malpractice liability, and/or anxiety of the patient. In this review, guidelines concerning the approach to some of the more common incidental cystic pancreatic lesions including IPMN, SPT (SPEN), serous cystadenoma, mucinous cystic tumor, as well as primary and secondary cystic splenic lesions will be presented.

